# Cross-Sectional Content Evaluation of Spinal Cord Injury Medicine Fellowship Websites

**DOI:** 10.7759/cureus.36183

**Published:** 2023-03-15

**Authors:** Frass Ahmed, Bilal Ali, Mahfujul Z Haque, Inaam Mohammed, Yusef Bazzy

**Affiliations:** 1 Medicine, Michigan State University College of Human Medicine, East Lansing, USA; 2 Physical Medicine and Rehabilitation, Oakland University William Beaumont School of Medicine, Auburn Hills, USA; 3 Physical Medicine and Rehabilitation, Wayne State University School of Medicine, Detroit, USA; 4 Physical Medicine and Rehabilitation, Wayne State University Detroit Medical Center, Detroit, USA

**Keywords:** prospective applicants, program, criteria, content, websites, fellowship, physical medicine & rehabilitation, spinal cord injury medicine

## Abstract

This study evaluated the quality and accessibility of the websites of the Spinal Cord Injury Medicine (SCIM) fellowship programs to identify potential areas for improvement for future applicants. Twenty-four SCIM fellowship program websites were analyzed based on 44 predetermined criteria: website accessibility, education, research, recruitment, and incentives. This study found that many evaluated websites needed more information on didactics, educational resources, evaluation criteria, call requirements, schedules, and expected caseloads, which could lead to an incomplete understanding of the fellowship program. Additionally, more information on education and research can be needed for applicants to adequately compare programs and make informed decisions about which programs to apply to. Information about the selection process, current board pass rates, mentorship opportunities, technology/simulation, and alumni was limited across several evaluated websites. Incentives, fellow wellness, and harassment policies were also found to be insufficient or absent. The study emphasizes that SCIM fellowship programs should provide comprehensive and accurate information on their websites to facilitate applicants in choosing the program that aligns best with their professional goals. Including detailed and accurate information regarding general program qualities, educational and research opportunities, recruitment, and incentive data will provide prospective applicants with a holistic understanding of the program. By providing detailed and transparent information on their websites, SCIM fellowships can attract more qualified applicants and enhance the applicant pool, ultimately improving the quality of their program.

## Introduction

Physical Medicine and Rehabilitation, commonly known as PM&R, offers various unique fellowship training experiences. One such discipline is known as spinal cord injury medicine (SCIM). PM&R residents and prospective SCIM fellowship applicants often utilize a fellowship program's website to learn about and choose between programs. While applicants are welcome to seek guidance from mentors or colleagues when selecting a program, the fellowship's website is regarded as the main source of information on which prospective fellows should base their decision [1]. The ease of access to information regarding a program aids prospective applicants in deciding whether to apply and whether a fellowship program suits their career goals and objectives [1-3]. When deciding on a program, applicants often consider several key factors, such as curriculum, rotations, compensation, benefits, and research opportunities, typically available on a program's website [1-3]. The importance of website information was further emphasized during the pandemic, as many applicants relied upon online resources to guide their decision while face-to-face consultation was limited [4]. As more applicants begin to rely upon program websites for information, the concern becomes whether such websites contain the appropriate amount and types of information for applicants to make an informed decision. Numerous studies have demonstrated that residency or fellowship websites commonly need more information to meet the needs of applicants [1,2,5]. Although current research highlights program website deficiencies in various specialties, studies have yet to examine the quality of the content of SCIM fellowship websites to date. This study aimed to provide a quantitative evaluation of SCIM fellowship websites to identify potential areas of improvement that can aid future fellowship applicants.

## Materials and methods

A cross-sectional analysis of 24 SCIM fellowship websites listed on the Fellowship Residency Electronic Interactive Database (FREIDA) on December 2022 was evaluated based on 44 predetermined criteria, including quantitative and descriptive statistics within four categories: website accessibility from FREIDA, education and research, recruitment, and incentives [6]. The authors read a series of previously published research studies evaluating website content for residency and fellowship programs and determined the 44 most commonly sought-after information applicants seek when searching for programs [1-3,5,7-9]. The criteria refer to the factors that interested applicants typically utilize in deciding their fellowship choices. Websites were accessed through the link provided by FREIDA. If the link needed to be fixed or directed to a program, not of interest, the website was searched through Google. A variable was considered included if its presence was on the department's website or FREIDA. The data was collected individually by two authors to ensure accuracy.

## Results

**Figure 1:**
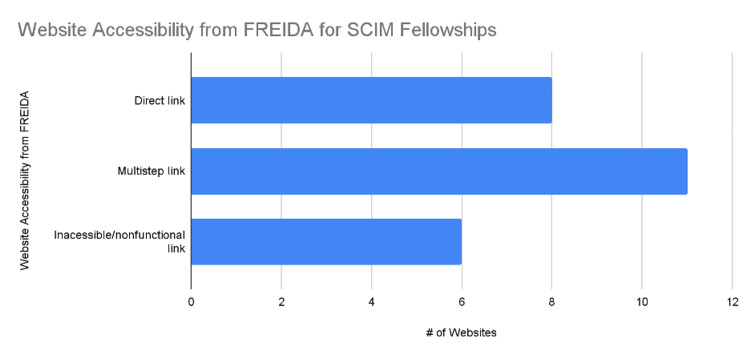
Website Accessibility from FREIDA for SCIM Fellowships FREIDA: Fellowship Residency Electronic Interactive Database; SCIM: Spinal Cord Injury Medicine

At the time of analysis, on December 2022, 24 active spinal cord injury medicine fellowship programs were identified [6]. These programs included: the University of California Irvine, Stanford University, the University of Colorado, the University of Miami, the University of South Florida Morsani, Northwestern University, the University of Louisville, Harvard University, Johns Hopkins University, the University of Michigan, the University of Minnesota, Rutgers University, Icahn Mount Sinai-NY, State University of New York (SUNY) Upstate, Case Western University, Pennsylvania State University, Thomas Jefferson University, the University of Pittsburgh, Baylor University, the University of Texas at Houston, the University of Utah, Virginia Commonwealth University, the University of Washington, and the Medical College of Wisconsin [6]. An analysis of website accessibility from FREIDA showed that 33% (8/24) of programs had a direct link to the SCIM fellowship page, whereas 46% (11/24) of programs provided a link to the departmental website (Figure 1). Of the remaining programs, 21% (5/24) provided a nonfunctional link (Figure 1).

**Figure 2:**
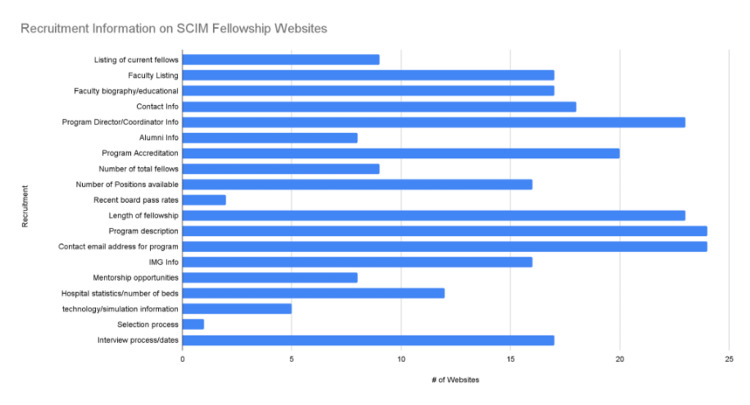
Recruitment Information on SCIM Fellowship Websites SCIM: Spinal Cord Injury Medicine

**Figure 3:**
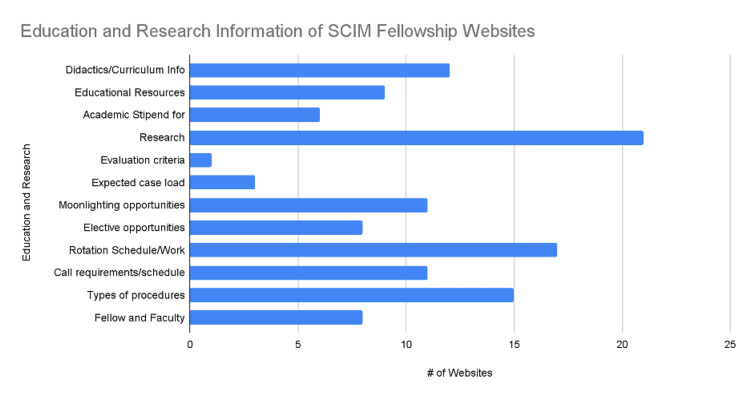
Education and Research Information of SCIM Fellowship Websites SCIM: Spinal Cord Injury Medicine

**Figure 4:**
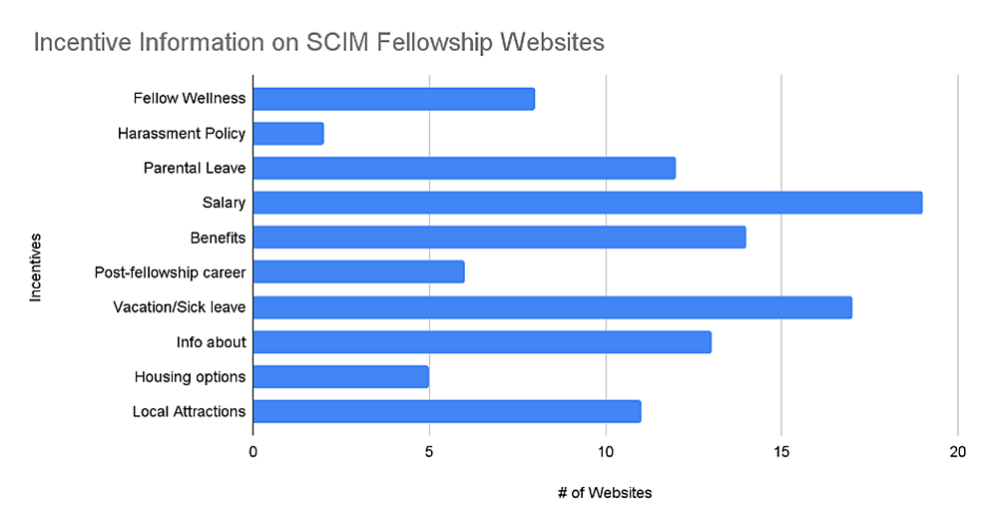
Incentive Information on SCIM Fellowship Websites SCIM: Spinal Cord Injury Medicine

All program websites (24/24, or 100%) contained a description of the program and a contact email address (Figure 2). There were two criteria that only one program website was able to provide: the selection process and the evaluation criteria (1/24, 4%) (Figure 2). In the education and research category, the criteria with the most accessibility on a program's website were research opportunities or requirements with 88% (21/24), followed by rotation schedule and work hours with 71% (17/24) (Figure 3). One program's website only reported evaluation criteria (Figure 3). Information regarding the expected caseload was only shared on 13% (3/24) of program websites (Figure 3). Regarding recruitment, the program director or coordinator's contact info was included in 96% (23/24) of websites. In comparison, the selection process and current board pass rates were reported at only 4% (1/24) and 8% (2/24), respectively (Figure 2). Regarding incentives for prospective applicants, the salary was mentioned on 79% (19/24) of the program's websites. The harassment policy was only mentioned twice out of the 24 program websites (8%) (Figure 4). 

Based on the evaluation, it was found that SCIM fellowship websites needed to be improved in several ways that applicants typically utilize to decide whether to apply for a fellowship (Table 1). Criteria that were identified as being under the 50% mark included education resources available to fellows, research stipend, evaluation criteria, fellow caseload, moonlighting and elective opportunities, board pass rates, info for international medical graduates, mentorship opportunities, wellness for fellows, information about recent career options from previous fellows, housing options, and local attractions to name a few (Table 1). A Cohen's kappa was conducted to determine the agreement between the website evaluators. It was determined that the evaluators had a near-perfect agreement with a K= 0.83. 

**Table 1:** Website Information on Spinal Cord Injury Medicine Fellowship Programs, n=24

Evaluation Criteria	Programs Fulfilling Criteria	Percentage
Direct Link	8	33%
Multistep Link	11	46%
Inaccessible/nonfunctional link	6	25%
Didactics/Curriculum Info	12	50%
Educational Resources Available to Fellows	9	38%
Academic Stipend for Research	6	25%
Research Opportunities/Requirements	21	88%
Evaluation Criteria	1	4%
Expected Case Load	3	13%
Moonlighting Opportunities	11	46%
Elective Opportunities	8	33%
Rotation Schedule/Work Hours	17	71%
Call Requirements/Schedule	11	46%
Description of Types of Procedures	15	63%
Fellow and Faculty Publications	8	33%
Listing of Current Fellows	9	37.50%
Faculty Listing	17	71%
Faculty Biography/Educational Background	17	71%
Faculty Contact Information	18	75%
Program Director/Coordinator Information	23	96%
Alumni Information	8	33%
Program Accreditation	20	83%
Number of Total Fellows	9	37.50%
Number of Positions Available	16	67%
Recent Board Pass Rates	2	8%
Length of Fellowship	23	96%
Program Description	24	100%
Contact Email Address for Program	24	100%
International Medical Graduate Information	16	67%
Mentorship Opportunities	8	33%
Hospital Statistics/Number of Beds	12	50%
Technology/Simulation of Information	5	21%
Selection Process	1	4%
Interview Process/Dates	17	71%
Fellow Wellness	8	33%
Harassment Policy	2	8%%
Parental Leave Mentioned	12	50%
Salary	19	79%
Benefits	14	58%
Post-Fellowship Career Description	6	25%
Vacation/Sick Leave	17	71%
Information about City/Community	13	54%
Housing Options	5	21%
Local Attractions	11	46%

## Discussion

Through a cross-sectional analysis of SCIM fellowship websites, this study focused on the presence or absence of key variables that can help applicants make informed decisions between programs of their choice. Many evaluated websites needed more information on didactics, educational resources, evaluation criteria, call requirements/schedule, and expected caseloads, which could lead to an incomplete understanding of the fellowship program. When such crucial information is unavailable, prospective applicants may need help understanding the objectives and expectations of a fellowship program. This lack of information could lead the applicants to apply for programs that do not align with their interests and career aspirations. It may also pose challenges for applicants to prepare for the program's rigors without having scheduling details. Furthermore, more information on education and research can be needed for applicants to adequately compare programs and make informed decisions about which programs to apply to or accept.

Several evaluated websites needed information on selection, current board pass rates, mentorship opportunities, technology and simulation resources, and alums. The information about the selection process is vital for applicants to tailor their application materials to meet the program's selection criteria, which can impact their chances of being accepted into the program. The current board passes rates provide applicants insight into the program's quality and the graduates' success rate. Similarly, applicants need more alum information to gauge the program's effectiveness and career advancement opportunities after completion. Information on mentorship opportunities, technology/simulation, and expected caseloads is valuable for applicants to evaluate the program's quality and make informed decisions about their personal and professional development. Therefore, comprehensive and accurate information on recruitment variables should be provided on a fellowship's website to facilitate applicants in choosing the program that aligns best with their professional goals.

Key information about incentives, fellow wellness, and harassment policies was insufficient or absent. These areas are integral to the decision-making process of prospective fellows. They should be a priority for fellowship programs to address to provide transparent and equitable opportunities to potential applicants [1-3]. Incentives such as benefits, compensation, and housing options are paramount to prospective fellows selecting a program [1-3]. Benefits such as health insurance, retirement plans, and vacation time enable fellows to balance their professional and personal lives.

Similarly, compensation is essential to a fellow's financial security and stability throughout their training. Housing options are also a significant consideration for applicants who may be relocating for the fellowship program. The absence of such information may disadvantage applicants who require comprehensive details of these incentives before committing to a program. Moreover, fellowship programs must ensure their fellows' well-being and safety by providing resources and programs that promote wellness and address physical and mental health concerns. The absence of information on fellow wellness policies and resources may hinder prospective applicants from making an informed decision. It may lead to a mismatch between the expectations and reality of their work environment. This is particularly challenging for those applicants who may be relocating, where the availability of such resources may be unfamiliar.

Similarly, the absence of information on harassment policies and procedures can create an environment that perpetuates harassment and negatively impacts the well-being and performance of fellows. Effective policies and procedures that address harassment and discrimination are essential for fostering a professional and safe work environment. Without this, prospective fellows may not feel comfortable reporting incidents and may be hesitant to apply to a program with a culture that is not conducive to addressing harassment effectively.

## Conclusions

Nearly all SCIM fellowship websites can provide prospective applicants with more detailed and transparent information. Accessibility to information is highly critical, especially during the current COVID-19 pandemic. As many fellowship interviews remain within the virtual space out of concerns for safety and feasibility, online platforms and their information are even more critical. Fellowship programs continuously strive to improve their processes to meet the diverse needs of applicants. This analysis of fellowship program websites is valuable for identifying improvement areas to enhance the match process's transparency and equity. Including detailed and accurate information regarding general program qualities, educational and research opportunities, recruitment, and incentive data will provide prospective applicants with a holistic understanding of the program. This can further assist applicants in making informed decisions about selecting a SCIM fellowship program that best aligns with their career objectives, which can improve the diversity of the fellows' cohort and ultimately elevate the quality of current programs.
